# Supplementary material for: Veteran and first responder family members show distinct mental health networks centered on negative emotions
Source: Commun Psychol. 2025 Aug 8;3:121. doi: 10.1038/s44271-025-00307-5 (PMC12334751; doi:10.1038/s44271-025-00307-5)
Supplement: Supplementary file 2 — Supplementary Fig. S1 [file 44271_2025_307_MOESM2_ESM.pdf]

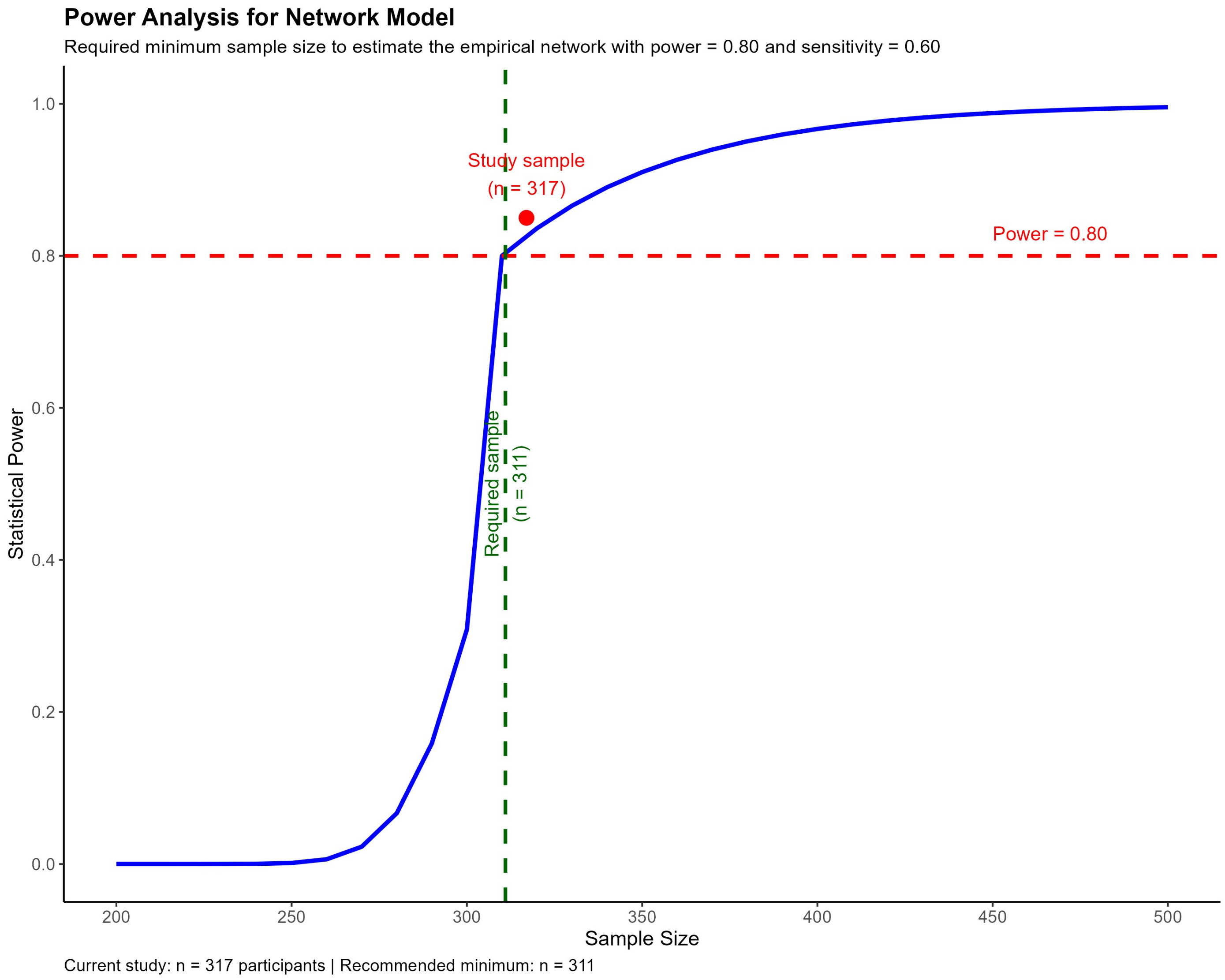

Supplementary Figure S1. Statistical power as a function of sample size.
Legend: Required minimum sample size to estimate the empirical network with a power of 0.80 and a sensitivity of 0.60 based on drawing 100 samples from a range of 200 to 500 with 500 bootstraps per sample.
