# Supplementary material for: Veteran and first responder family members show distinct mental health networks centered on negative emotions
Source: Commun Psychol. 2025 Aug 8;3:121. doi: 10.1038/s44271-025-00307-5 (PMC12334751; doi:10.1038/s44271-025-00307-5)
Supplement: Supplementary file 3 — Reporting summary [file 44271_2025_307_MOESM3_ESM.pdf]

nature portfolio

Corresponding author(s): Oleg N. Medvedev

Last updated by author(s): 11/07/2025

Reporting Summary

Nature Portfolio wishes to improve the reproducibility of the work that we publish. This form provides structure for consistency and transparency in reporting. For further information on Nature Portfolio policies, see our Editorial Policies and the Editorial Policy Checklist.

Statistics

For all statistical analyses, confirm that the following items are present in the figure legend, table legend, main text, or Methods section.

n/a	Confirmed	
☐	☒	The exact sample size (*n*) for each experimental group/condition, given as a discrete number and unit of measurement
☐	☒	A statement on whether measurements were taken from distinct samples or whether the same sample was measured repeatedly
☐	☒	The statistical test(s) used AND whether they are one- or two-sided<br>*Only common tests should be described solely by name; describe more complex techniques in the Methods section.*
☐	☒	A description of all covariates tested
☐	☒	A description of any assumptions or corrections, such as tests of normality and adjustment for multiple comparisons
☐	☒	A full description of the statistical parameters including central tendency (e.g. means) or other basic estimates (e.g. regression coefficient) AND variation (e.g. standard deviation) or associated estimates of uncertainty (e.g. confidence intervals)
☐	☒	For null hypothesis testing, the test statistic (e.g. *F*, *t*, *r*) with confidence intervals, effect sizes, degrees of freedom and *P* value noted<br>*Give P values as exact values whenever suitable.*
☒	☐	For Bayesian analysis, information on the choice of priors and Markov chain Monte Carlo settings
☐	☒	For hierarchical and complex designs, identification of the appropriate level for tests and full reporting of outcomes
☐	☒	Estimates of effect sizes (e.g. Cohen's *d*, Pearson's *r*), indicating how they were calculated

Our web collection on statistics for biologists contains articles on many of the points above.

Software and code

Policy information about availability of computer code

Data collection	No software were used.
Data analysis	The network model estimated in this study was computed using R (version 4.4.0), and the code is available as a supplementary file.

For manuscripts utilizing custom algorithms or software that are central to the research but not yet described in published literature, software must be made available to editors and reviewers. We strongly encourage code deposition in a community repository (e.g. GitHub). See the Nature Portfolio guidelines for submitting code & software for further information.

Data

Policy information about availability of data

All manuscripts must include a data availability statement. This statement should provide the following information, where applicable:

- Accession codes, unique identifiers, or web links for publicly available datasets
- A description of any restrictions on data availability
- For clinical datasets or third party data, please ensure that the statement adheres to our policy

The data are from a treatment-seeking group of family members who consented to clinical treatment and used it in possible research studies. However, they did not give permission for their data to be open-access.

Research involving human participants, their data, or biological material

Policy information about studies with human participants or human data. See also policy information about sex, gender (identity/presentation), and sexual orientation and race, ethnicity and racism.

Reporting on sex and gender	Most of the participants (82%) were women as vast majority of first responders and veterans were men, which did not permit meaningful statistical analyses of sex differences between family members. This is acknowledged as a limitation.
Reporting on race, ethnicity, or other socially relevant groupings	We used the following socially constructed/socially relevant categorization variables in our manuscript:<br>Gender: Used to describe participant demographics (82.0% women, 17.7% men, 0.3% non-binary). Gender was ascertained through self-report during intake assessment. This variable was used for descriptive purposes to characterize our sample and was not used as a proxy for other socially constructed variables or biological sex.<br>Race/Ethnicity: Used to describe participant demographics including African American/Black (9.1%), Asian American (2.5%), Latino(a)/Hispanic (12.4%), Multiple Ethnicities (2.5%), Native American (1.6%), White (71.3%), and Hawaiian/Pacific Islander (0.6%). Race and ethnicity data were collected through participant self-report during intake assessment. These variables were used solely for sample description and demographic characterization, not as proxies for other social constructs.Clear definitions: Gender and race/ethnicity categories were defined by participant self-identification during clinical intake. We did not control for these variables as confounding variables in our network analyses, as our analytical approach (network analysis using Gaussian graphical models) focused on symptom-level relationships rather than group comparisons by demographic characteristics.<br>Method for classification: All demographic data were obtained through standard clinical intake forms completed by participants via self-report.
Population characteristics	The study included 317 treatment-seeking family members of trauma-exposed veterans and first responders. Key characteristics include: average age 38.28 years (SD=12.44, range=54 years); 82.0% women, 17.7% men, 0.3% non-binary; 71.3% white, 12.4% Latino(a)/Hispanic, 9.1% African American/Black, 2.5% Asian American, 2.5% multiple ethnicities, 1.6% Native American, 0.6% Hawaiian/Pacific Islander; 65.7% completed some college or higher education; 58.7% married; 57.1% were significant others of veterans/first responders, 40.7% were family members, 2.2% were adult children; 65.3% were family of veterans, 34.7% were family of first responders. Past and current diagnoses were assessed using standardized clinical measures (PCL-5, PHQ-9, GAD-7) rather than formal diagnostic categories. No specific treatment categories were analyzed as this was a cross-sectional intake assessment study.
Recruitment	Participants were recruited from individuals seeking counseling services at a non-profit organization serving veterans, first responders, frontline healthcare workers, and their families between 2015-2021. This represents a convenience sample of treatment-seeking individuals, which introduces potential selection bias toward those with higher symptom severity and help-seeking behavior. The sample may not be representative of all family members of veterans and first responders, particularly those not seeking mental health services. Geographic bias may be present as participants were drawn from a single organization's catchment area. The high proportion of women (82%) may reflect gender differences in help-seeking behavior. These biases likely impact generalizability to non-treatment-seeking populations and may overestimate symptom prevalence and severity.
Ethics oversight	This study was approved by Stephen F. Austin State University Institutional Review Board, ensuring compliance with ethical standards for research involving human participants. All participants provided informed consent, and the study adhered to principles of inclusivity, transparency, and responsible data collection.

Note that full information on the approval of the study protocol must also be provided in the manuscript.

Field-specific reporting

Please select the one below that is the best fit for your research. If you are not sure, read the appropriate sections before making your selection.

☒ Life sciences ☐ Behavioural & social sciences ☐ Ecological, evolutionary & environmental sciences

For a reference copy of the document with all sections, see nature.com/documents/nr-reporting-summary-flat.pdf

Life sciences study design

All studies must disclose on these points even when the disclosure is negative.

Sample size	No formal statistical power analysis was conducted to predetermine sample size. The sample consisted of all eligible family members (n=317) who sought counseling services at the participating organization between 2015-2021 and met inclusion criteria (related to a first responder or veteran, over age 18, completed baseline assessments verified for completeness). This convenience sample size was deemed sufficient for network analysis based on established guidelines suggesting adequate stability for networks with our number of nodes, and our stability analyses confirmed adequate network reliability with correlation stability coefficient exceeding 0.59.
Data exclusions	Inclusion criteria were: being related to a first responder or veteran, over the age of 18, and completing baseline assessments verified for completeness by the intake manager. No participants meeting these criteria were excluded from analyses. All participants who completed the required measures (PCL-5, PHQ-9, GAD-7) were included in the final analytic sample (n=317).
Replication	This study did not include replication attempts as it represents a cross-sectional analysis of clinical intake data. However, we conducted extensive bootstrap analyses (1000 bootstrapped samples) to assess the stability and reliability of our network findings. The correlation

	stability coefficient (r=0.70) exceeded the recommended threshold of 0.59, indicating adequate network stability across resamples.
Randomization	This study did not involve experimental group allocation as it was an observational cross-sectional analysis of clinical intake data. Participants were not randomized or assigned to different conditions. All participants completed the same standardized assessment battery as part of routine clinical intake procedures.
Blinding	Investigator blinding was not applicable to this study as it involved secondary analysis of de-identified clinical intake data collected as part of routine care procedures. No experimental manipulation or group allocation occurred that would require blinding protocols.

Reporting for specific materials, systems and methods

We require information from authors about some types of materials, experimental systems and methods used in many studies. Here, indicate whether each material, system or method listed is relevant to your study. If you are not sure if a list item applies to your research, read the appropriate section before selecting a response.

Materials & experimental systems

n/a	Involved in the study
☒	☐ Antibodies
☒	☐ Eukaryotic cell lines
☒	☐ Palaeontology and archaeology
☒	☐ Animals and other organisms
☐	☒ Clinical data
☒	☐ Dual use research of concern
☒	☐ Plants

Methods

n/a	Involved in the study
☒	☐ ChIP-seq
☒	☐ Flow cytometry
☒	☐ MRI-based neuroimaging

Clinical data

Policy information about clinical studies

All manuscripts should comply with the ICMJE guidelines for publication of clinical research and a completed CONSORT checklist must be included with all submissions.

Clinical trial registration	Not applicable. This study was not a clinical trial but rather an observational cross-sectional analysis of clinical intake data from family members seeking mental health services. No intervention or treatment was tested.
Study protocol	No formal study protocol was preregistered as this was a secondary analysis of existing clinical intake data. The analytical approach involved network analysis and community detection of mental health symptoms using standardized clinical measures collected during routine care.
Data collection	Data were collected at intake from family members who sought counseling services between 2015-2021 at a non-profit organization serving veterans, first responders, frontline healthcare workers, and their families. Data collection occurred during participants' initial appointment before beginning treatment. Demographic data and standardized assessments (PCL-5, PHQ-9, GAD-7) were completed as part of routine clinical intake procedures at the organization's facilities.
Outcomes	This study did not have pre-defined primary and secondary outcome measures as it was an exploratory cross-sectional analysis rather than a hypothesis-testing study. The analytical outcomes involved: (1) identification of symptom networks and communities using network analysis, (2) assessment of network stability through bootstrap procedures, and (3) examination of node centrality measures. Mental health symptoms were assessed using validated clinical measures: PCL-5 for PTSD symptoms, PHQ-9 for depression symptoms, and GAD-7 for generalized anxiety symptoms.

Plants

Seed stocks	*Report on the source of all seed stocks or other plant material used. If applicable, state the seed stock centre and catalogue number. If plant specimens were collected from the field, describe the collection location, date and sampling procedures.*
Novel plant genotypes	*Describe the methods by which all novel plant genotypes were produced. This includes those generated by transgenic approaches, gene editing, chemical/radiation-based mutagenesis and hybridization. For transgenic lines, describe the transformation method, the number of independent lines analyzed and the generation upon which experiments were performed. For gene-edited lines, describe the editor used, the endogenous sequence targeted for editing, the targeting guide RNA sequence (if applicable) and how the editor was applied.*
Authentication	*Describe any authentication procedures for each seed stock used or novel genotype generated. Describe any experiments used to assess the effect of a mutation and, where applicable, how potential secondary effects (e.g. second site T-DNA insertions, mosiacism, off-target gene editing) were examined.*
